# Supplementary material for: The causal relationship between the human gut microbiota and pyogenic arthritis: a Mendelian randomization study
Source: Front Cell Infect Microbiol. 2024 Nov 26;14:1452480. doi: 10.3389/fcimb.2024.1452480 (PMC11629706; doi:10.3389/fcimb.2024.1452480)
Supplement: Supplementary file 1 [file DataSheet1.zip › mendelian test/ebi-a-GCST90027598.csv_leave_one_out.pdf]

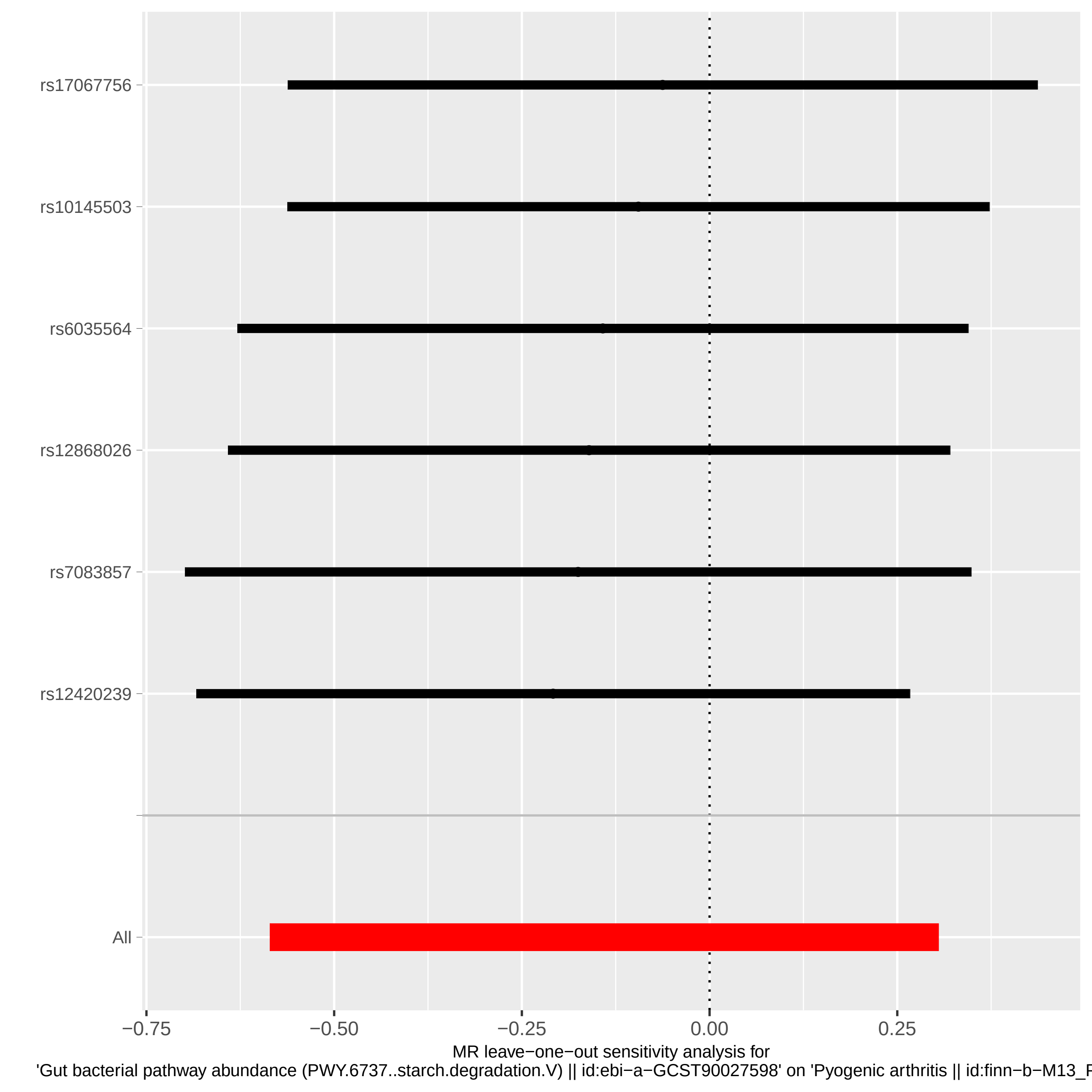
rs17067756
rs10145503
rs6035564
rs12868026
rs7083857
rs12420239
All
−0.75
−0.50
−0.25
0.00
0.25
MR leave−one−out sensitivity analysis for
'Gut bacterial pathway abundance (PWY.6737..starch.degradation.V) || id:ebi−a−GCST90027598' on 'Pyogenic arthritis || id:finn−b−M13_P
